# Assessment of myocardial oxygenation in patients with severe aortic stenosis before and after aortic valve replacement: an oxygenation-sensitive CMR study

**DOI:** 10.1186/1532-429X-16-S1-M12

**Published:** 2014-01-16

**Authors:** Masliza Mahmod, Jane M Francis, Nikhil Pal, Andrew Lewis, Sairia Dass, Ravi De Silva, Mario Petrou, Rana Sayeed, Stephen Westaby, Matthew D Robson, Houman Ashrafian, Stefan Neubauer, Theodoros D Karamitsos

**Affiliations:** 1 Oxford Centre for Clinical Magnetic Resonance Research, Division of Cardiovascular Medicine, Radcliffe Department of Medicine, Oxford, UK; 2 Department of Cardiothoracic Surgery, Oxford University Hospitals, Oxford, UK

## Background

Left ventricular hypertrophy in aortic stenosis (AS) is characterised by reduced myocardial perfusion reserve due to coronary microvascular dysfunction. However, it remains unclear whether the hypoperfusion seen in severe AS leads to myocardial tissue deoxygenation and thus, ischemia during stress. The aim of this study was to assess myocardial oxygenation and perfusion in patients with severe AS but no obstructive coronary artery disease before and after aortic valve replacement (AVR).

## Methods

Twenty two patients with isolated severe AS and unobstructed epicardial coronary arteries on invasive coronary angiography planned to undergo AVR were prospectively recruited. All patients underwent cardiovascular magnetic resonance (CMR) at 3 Tesla. Myocardial function, perfusion (myocardial perfusion reserve index - MPRI) and oxygenation (blood-oxygen level dependent-BOLD signal intensity - SI change) during adenosine stress and rest, and fibrosis (late gadolinium enhancement - LGE) were assessed. Of the 22 patients who had AVR, 10 of them were rescanned at 8.0 ± 2.1 months after AVR. Fifteen age- and gender-matched healthy volunteers served as controls.

## Results

**Figure 1:**
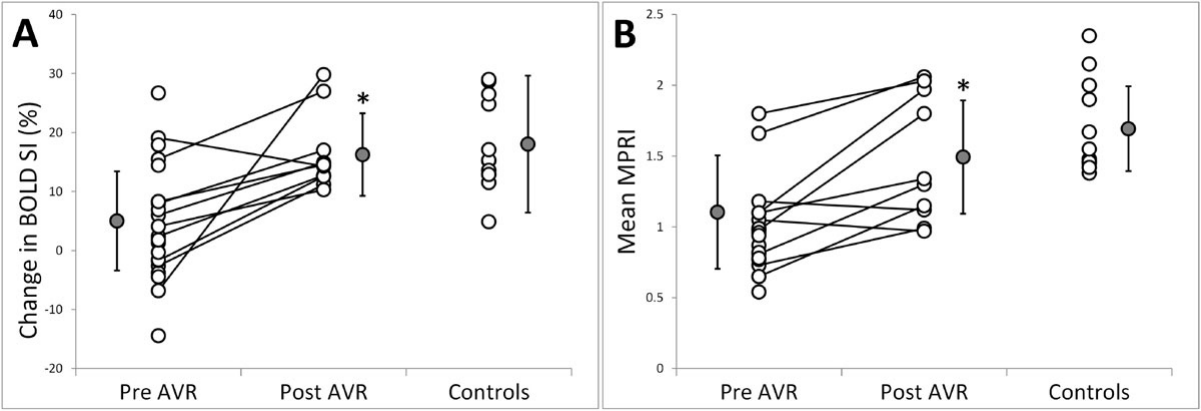
**BOLD SI Change and MPRI before and after AVR**.

Clinical characteristics and CMR are presented in Table 1. All subjects were matched for age, gender and body mass index. AS patients had reduced perfusion reserve (MPRI 1.0 ± 0.3 vs. controls 1.7 ± 0.3, p < 0.001) and blunted oxygenation response during stress (BOLD SI change 4.8 ± 9.6% vs. controls 18.2 ± 11.6%, p = 0.001). Myocardial perfusion and oxygenation showed a positive correlation (R = 0.48, p = 0.005). Both MPRI and BOLD had an inverse correlation with maximal left ventricular (LV) wall thickness, R = -0.71, p < 0.001 and R = -0.46, p = 0.005, respectively. Myocardial perfusion (R = -0.42, p = 0.02) but not oxygenation (R = -0.4, p = 0.06) significantly correlated with myocardial fibrosis, respectively. There was significant regression of LVH after AVR (LVMI from 100 ± 36 g/m2 to 71 ± 19 g/m2 and LV wall thickness from 16 ± 2 mm to 14 ± 2 mm) although LVMI and LV wall thickness were still significantly higher compared to normal controls (p < 0.05 vs. pre AVR and p < 0.05 vs. controls). Importantly, there was substantial improvement in perfusion and oxygenation towards normal after AVR, MPRI 1.5 ± 0.4, p = 0.005 vs. 1.1 ± 0.4 pre AVR and BOLD SI change 16.4 ± 7.0%, p = 0.014 vs. 4.9 ± 8.4% pre AVR (Figure 1).

**Table 1:** Baseline clinical and CMR findings

	Severe AS (n = 22)	Normal controls (n = 15)	P value
Age (years)	67 ± 9	63 ± 4	0.09
Male, n (%)	15 (68)	8 (53)	0.34
Body mass index (kg/m2)	27 ± 4	26 ± 3	0.38
BOLD signal intensity change (%)	4.8 ± 9.6	18.2 ± 11.6	0.001
Myocardial perfusion reserve index	1.0 ± 0.3	1.7 ± 0.3	< 0.001
Left ventricular ejection fraction (%)	74 ± 6	69 ± 4	0.01
Left ventricular end-diastolic volume (ml)	148 ± 46	142 ± 32	0.65
Left ventricular end-systolic volume (ml)	40 ± 20	44 ± 12	0.43
Left ventricular wall thickness (mm)	17 ± 3	10 ± 1	< 0.001
Left ventricular mass index (g/m2)	99 ± 38	56 ± 13	< 0.001
Aortic valve area (cm2)	0.84 ± 0.10	-	-
LGE present, n (%)	16 (73)	0 (0)	-
LGE volume when positive (%)	17.6 ± 13.8	-	-

Values are mean SD or percentages. BOLD, blood oxygen level dependent; LGE, late gadolinium enhancement.

## Conclusions

In severe AS without epicardial CAD, there is blunted oxygenation response to adenosine stress suggestive of microvascular impairment. Myocardial perfusion and oxygenation are restored following AVR. Oxygenation-sensitive CMR provides pathophysiologic insight, may become a helpful diagnostic tool, and suggests novel strategies for therapy in AS aimed at improving the oxygen demand/supply balance.

## Funding

None.

